# Helium Conditioning Increases Cardiac Fibroblast Migration Which Effect Is Not Propagated via Soluble Factors or Extracellular Vesicles

**DOI:** 10.3390/ijms221910504

**Published:** 2021-09-29

**Authors:** Marek Jelemenský, Csenger Kovácsházi, Kristína Ferenczyová, Monika Hofbauerová, Bernadett Kiss, Éva Pállinger, Ágnes Kittel, Viktor Nabil Sayour, Anikó Görbe, Csilla Pelyhe, Szabolcs Hambalkó, Lucia Kindernay, Miroslav Barančík, Péter Ferdinandy, Monika Barteková, Zoltán Giricz

**Affiliations:** 1 Institute for Heart Research, Centre of Experimental Medicine, Slovak Academy of Sciences, 84104 Bratislava, Slovakia; marek.jelemensky@savba.sk (M.J.); kristina.ferenczyova@savba.sk (K.F.); lucia.griecsova@savba.sk (L.K.); miroslav.barancik@savba.sk (M.B.); 2 Department of Pharmacology and Pharmacotherapy, Semmelweis University, 1089 Budapest, Hungary; kovacshazi.csenger@med.semmelweis-univ.hu (C.K.); kiss.bernadett@med.semmelweis-univ.hu (B.K.); sayour.viktor_nabil@med.semmelweis-univ.hu (V.N.S.); gorbe.aniko@med.semmelweis-univ.hu (A.G.); pelyhe.csilla@med.semmelweis-univ.hu (C.P.); hambalko.szabolcs@med.semmelweis-univ.hu (S.H.); peter.ferdinandy@pharmahungary.com (P.F.); 3 Institute of Physics, Slovak Academy of Sciences, Dúbravská Cesta 9, 84511 Bratislava, Slovakia; monika.hofbauerova@savba.sk; 4 Centre for Advanced Materials Application, Slovak Academy of Sciences, Dúbravská Cesta 9, 84511 Bratislava, Slovakia; 5 MTA-SE System Pharmacology Research Group, Department of Pharmacology and Pharmacotherapy, Semmelweis University, 1089 Budapest, Hungary; 6 Department of Genetics, Cell and Immunobiology, Semmelweis University, 1089 Budapest, Hungary; eva.pallinger@gmail.com; 7 Institute of Experimental Medicine, Eötvös Loránd Research Network, 1083 Budapest, Hungary; kittel.agnes@koki.hu; 8 Pharmahungary Group, 6722 Szeged, Hungary; 9 Institute of Physiology, Faculty of Medicine, Comenius University in Bratislava, 81372 Bratislava, Slovakia

**Keywords:** helium conditioning, fibroblast, NRCF, endothelial cell, HUVEC, migration, angiogenesis, extracellular vesicles, microvesicles, heart

## Abstract

Helium inhalation induces cardioprotection against ischemia/reperfusion injury, the cellular mechanism of which remains not fully elucidated. Extracellular vesicles (EVs) are cell-derived, nano-sized membrane vesicles which play a role in cardioprotective mechanisms, but their function in helium conditioning (HeC) has not been studied so far. We hypothesized that HeC induces fibroblast-mediated cardioprotection via EVs. We isolated neonatal rat cardiac fibroblasts (NRCFs) and exposed them to glucose deprivation and HeC rendered by four cycles of 95% helium + 5% CO_2_ for 1 h, followed by 1 h under normoxic condition. After 40 h of HeC, NRCF activation was analyzed with a Western blot (WB) and migration assay. From the cell supernatant, medium extracellular vesicles (mEVs) were isolated with differential centrifugation and analyzed with WB and nanoparticle tracking analysis. The supernatant from HeC-treated NRCFs was transferred to naïve NRCFs or immortalized human umbilical vein endothelial cells (HUVEC-TERT2), and a migration and angiogenesis assay was performed. We found that HeC accelerated the migration of NRCFs and did not increase the expression of fibroblast activation markers. HeC tended to decrease mEV secretion of NRCFs, but the supernatant of HeC or the control NRCFs did not accelerate the migration of naïve NRCFs or affect the angiogenic potential of HUVEC-TERT2. In conclusion, HeC may contribute to cardioprotection by increasing fibroblast migration but not by releasing protective mEVs or soluble factors from cardiac fibroblasts.

## 1. Introduction

Cardiovascular diseases (CVDs) represent the leading cause of deaths in the modern world, accounting for over 4 million deaths per year in European countries. Specifically, ischemic heart disease (IHD) is the most prevalent cause of death from CVDs with 38% of all CVD-related deaths in females and 44% of deaths in males [1]. Despite advances in the current pharmacological and non-pharmacological therapies of IHD, this disease is still a leading cause of mortality and hospitalization worldwide. Thus, there is still an unmet need for improving relevant therapies. The cardiac tissue is mainly composed of cardiomyocytes (CMs), endothelial cells (ECs), and cardiac fibroblasts (CFs) [2]. Although CMs are the cells primarily responsible for the cardiac pump function, ECs and CFs are of key importance in maintaining the appropriate milieu for CMs. CFs and ECs are heavily involved in post myocardial infarction (MI) healing, as well as scar formation and remodeling. During the healing phase of MI, CFs migrate into the scar, undergo rapid phenotypic changes, and transform to myofibroblasts, which have the phenotype of both fibroblasts and smooth muscle cells. They are necessary for the production of extracellular matrix after MI to avoid rupture of the ventricular wall, an acute and fatal mechanical complication of MI [3,4,5,6]. CFs are also involved in the protection of cardiomyocytes against sustained MI damage via paracrine factors [7,8,9]. Meanwhile, neoangiogenesis is crucial to restore blood supply after MI [10]. Therefore, modulating the function of CFs or ECs may lead to improved cardioprotective therapies.

One of the most promising therapies for IHD is remote ischemic conditioning, first described by Przyklenk et al. [11], however its clinical benefit has not yet been proven [12,13,14,15]. Therefore, novel interventions and therapeutic strategies are needed for the treatment of IHD [16,17]. To this end, conditioning with helium (HeC) or other inert gases has been investigated. It was proven that they reduce infarct size in rodents and rabbits [18,19,20]. However, the molecular effect of HeC on the cardiac fibroblasts and its mechanism of action is still unknown.

Extracellular vesicles (EVs) are small, lipid membrane particles released by most mammalian cells. They are mostly defined by their size. EVs under 150 nm in diameter are identified as small EVs (sEVs), between 150 and 1000 nm as medium EVs (mEVs), and over 1000 nm as large EVs [21]. They are involved in intercellular communication, and they have an important role in IHD and cardioprotection [22]. EVs secreted by CMs can activate CFs after ischemia [23,24,25]. On the other hand, EVs released from CFs after ischemia may protect CMs against ischemia-induced cell death [26]. Moreover, we have shown that EVs play key role in ischemic conditioning [27]. These results support that EVs are important in the stress-adaptive mechanisms of the heart. Thus, they can play a role in HeC as well. Weber et al. have shown that HeC increased caveolin-3 secretion in sEVs [28]. However, Smit et al. did not find any change in EV secretion in volunteers who received HeC before forearm ischemia [29]. According to the contradicting literature and since that there is no information on EV release from CFs in HeC, new research in the field is needed to uncover the possible EV-based cardioprotective signaling pathways of HeC. 

Therefore, we have set up an in vitro model of HeC on CFs. We have tested whether HeC-treated CFs secrete EVs or soluble factors which can activate fibroblasts or ECs. We have revealed that HeC may improve cardiac repair via the acceleration of CF migration. However, it does not induce myofibroblast transformation and does not activate fibroblasts and endothelial cells via CF mEVs or soluble factors.

## 2. Results

### 2.1. HeC Increases Migration of Cardiac Fibroblasts

We have isolated neonatal rat cardiac fibroblasts (NRCFs) and tested them with flow cytometry (FC) for viability, apoptosis, and for the expression of fibroblast markers. Our results have shown the high viability of cells with low signal for Annexin V, which indicates no apoptosis. Our cells also expressed common fibroblast markers Thy-1 and DDR2 (Supplementary Figure S1).

**Figure 1:**
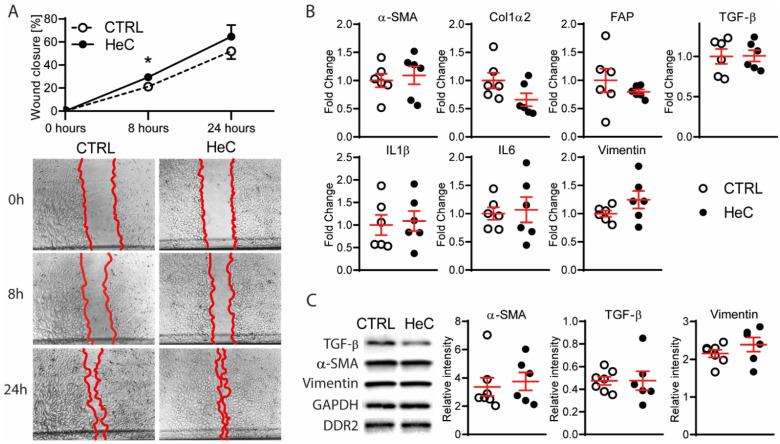
HeC induces fibroblast migration, but not myofibroblast transformation or inflammation; (**A**) Migration of CTRL and HeC NRCFs after scratch stimuli with representative images 0, 8 and 24 h after scratch with 5× magnification; (**B**) qPCR analysis of markers of myofibroblast transformation (*α-SMA*, *Col1α2*, *FAP* and *TGF-β*) and inflammatory markers (*IL1β* and *IL6*) of CTRL and HeC NRCFs. Vimentin was used as a control for fibroblast phenotype; (**C**) WB representative bands and relative protein expression of α-SMA and Vimentin compared to DDR2 and TGF-β compared to GAPDH in CTRL and HeC NRCF cells; n = 4–7. Results are represented as mean ± SEM; * *p* < 0.05 vs. CTRL.

To investigate the potential effects of HeC on NRCFs, we measured their migration after conditioning. We found that HeC increased NRCF migration at 8 h significantly (Figure 1A). However, this effect was not significant at 24 h, suggesting that HeCs accelerate the migration of NRCFs temporarily.

Next, we analyzed if the increased cell migration is associated with the activation of fibroblasts. Using a quantitative polymerase chain reaction (qPCR), we tested alpha smooth muscle action (*α-SMA*) and collagen type 1 alpha 2 (*Col1α2*) as the most common genes up-regulated in myofibroblasts. Furthermore, we analyzed fibroblast activation protein (*FAP*), which is upregulated in several cardiac diseases but is not expressed at a significant level in healthy tissue [30]. Transforming growth factor beta (*TGF-β*) was also analyzed to test the feed-forward activation of fibroblasts, and *IL1β* and *IL6* were analyzed as the markers of inflammation. We found that none of the analyzed genes were differentially expressed due to HeC (Figure 1B), suggesting that HeC does not induce the activation, myofibroblast transformation, or pro-inflammatory processes of NRCFs. These results were validated using Western blots (WB) for α-SMA and TGF-β (Figure 1C).

### 2.2. HeC Tends to Decrease mEV Secretion from NRCFs

We tested how HeC affects the mEV secretion from NRCFs. We set up our EV isolation protocol with H9C2 cardiac cell line derived EVs. We applied differential centrifugation to the cell supernatant with or without filtration. As filtration did not increase the purity of the isolate but might have reduced its yield (Supplementary Figure S2), we decided to exclude filtration from the final protocol.

**Figure 2:**
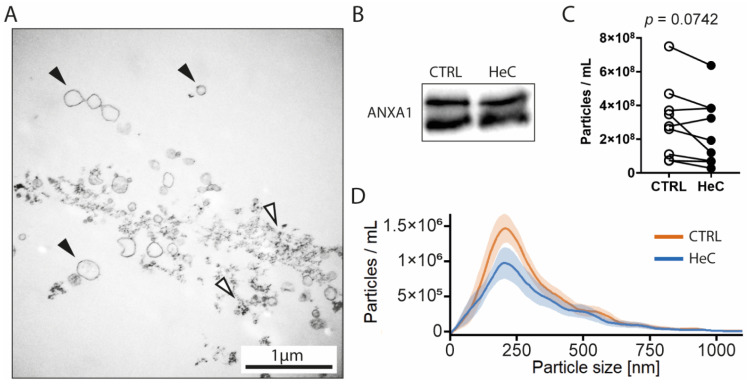
HeC tends to decrease NRCF mEV secretion: (**A**) EM image of mEV isolate, black arrow: membrane vesicles; white arrow: protein aggregates; (**B**) Qualitative analysis of Annexin A1 expression of mEVs with WB; (**C**) NTA concentration analysis of mEV isolates; (**D**) NTA size distribution analysis of mEV isolates. Results are represented as mean ± SEM.

Next, we isolated mEVs from NRCF cell supernatant 40 h after HeC. We have detected membrane particles with the expected size-range using transmission electron microscopy (TEM), but minor protein contamination was also visible (Figure 2A). Using WB, we proved that isolated particles are positive to EV cargo protein Annexin A1 (ANXA1) [21] but negative for Glyceraldehyde 3-phosphate dehydrogenase (GAPDH) that can accumulate in EVs [21]. Meanwhile, samples were negative to other cellular markers, such as ATP synthase lipid-binding protein (ATP5A), sodium-potassium ATPase (Na-K-ATPase), and Histone H3 (Figure 2B). We have tested isolated mEVs for fibroblast markers DDR2 and Thy-1 to prove that these vesicles were released from NRCFs. Our samples shown only a moderate presence of Thy-1 marker on their surface and very low expression of DDR2 (Supplementary Figure S3).

To analyze the size distribution and concentration of mEVs, we compared the resolution of dynamic light scattering (DLS) and nanoparticle tracking analysis (NTA). We found that neither of the methods was able to resolve the mixed population of calibration beads (Supplementary Figure S4B,C). However, as expected, NTA shown a broader size distribution for mEV samples, while DLS displayed only a single peak (Figure S4A). Therefore, we used NTA in our further experiments.

The particle size distribution of mEV by NTA was between 50 and 750 nm, which corresponds to the expected size range of mEVs. With the analysis of the total particle concentrations, we have revealed that HeC tends to decrease the mEV secretion from NRCFs regardless of the culturing volume of the cells, while the modal particle size remains unchanged (Figure 2C).

### 2.3. NRCFs Do Not Secrete Fibrotic or Angiogenic EVs or Other Factors Regardless to HeC Conditioning

**Figure 3:**
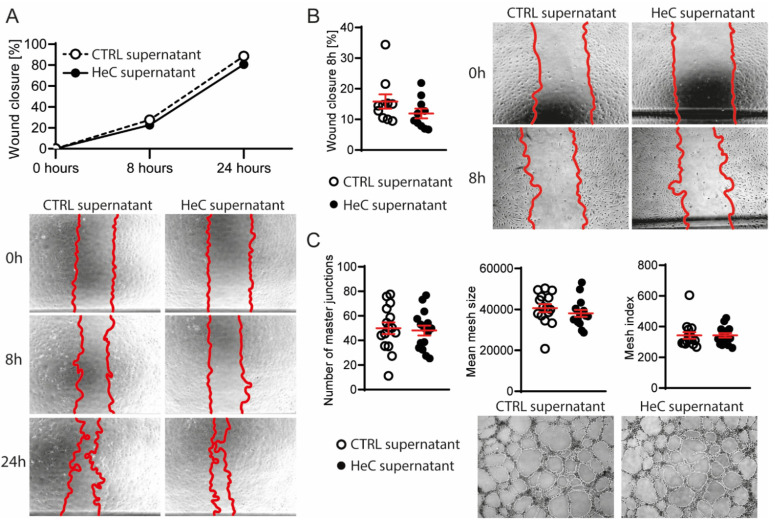
Effect of HeC is not transferred to naïve fibroblast or endothelial cells via soluble factors; (**A**) Migration of naïve NRCF cells after transfer of CTRL or HeC NRCF supernatant with representative images 0, 8 and 24 h after scratch with 5× magnification; (**B**) Migration of HUVEC-TERT2 treated with CTRL or HeC NRCF supernatant with representative images 0 and 8 h after scratch with 5× magnification; (**C**) In vitro angiogenesis of HUVEC-TERT2 treated with CTRL or HeC NRCF supernatant with representative images 6 h after treatment with 5× magnification. Number of master junctions represent the number of nodes, mean mesh size represent the mean size of the meshes, Mesh index represents the mean length of the branches. n = 4–15. Results are represented as mean ± SEM.

As EVs and other soluble factors can be important mediators of intercellular response to MI, we evaluated whether HeC treated NRCFs affect tissue remodeling via the activation of fibroblasts or ECs. To this end, we transferred the supernatant of control and HeC NRCFs to intact NRCFs or immortalized human umbilical vein endothelial cells (HUVEC-TERT2). The migration of naïve cells did not change after treatment (Figure 3A,B), which indicates that the HeC-induced migration of NRCFs is not transferred by EVs or any soluble factors. Moreover, the HeC NRCF supernatant did not affect the in vitro tube formation of HUVEC-TERT2 (Figure 3C).

## 3. Discussion

**Figure 4:**
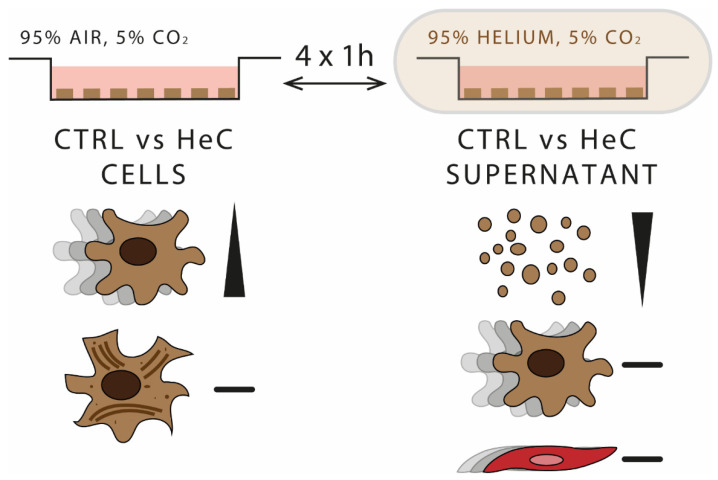
HeC accelerates fibroblast migration without inducing myofibroblast transformation but does not affect fibrosis and angiogenesis via secreted factors. In vitro HeC protocol composed of 4 times one hour 95% helium 5% CO_2_ followed by one hour 95% air 5% CO_2_ conditions. NRCF cells migrated faster after HeC treatment, but they did not transform to myofibroblasts. mEV concentration tended to decrease in HeC NRCF supernatant. Supernatant of HeC-treated NRCFs did not induce fibrosis or angiogenesis in naïve cells.

This is the first demonstration that HeC accelerated the migration of NRCFs, while it did not induce their transformation to myofibroblasts. Moreover, HeC tended to decrease the mEV secretion of NRCFs. However, the supernatant of HeC-treated NRCFs did not accelerate the migration of naïve NRCFs or HUVEC-TERT2 and did not induce angiogenesis. These results suggest that HeC may exert its cardioprotective effect via NRCFs but not via NRCF EVs or soluble factors (Figure 4).

As the mobilization of fibroblasts is needed in the early remodeling of the heart after MI [31], especially given that resident CFs are the major source of infiltrating fibroblasts during post-MI repair [32], our finding that HeC accelerated the migration of NRCFs can be considered beneficial for post-MI wound healing. This finding amends previous data on the beneficial effects of HeC which shown that HeC reduces infarct size in young rats [19]. Not only fibroblast infiltration, but the extracellular matrix production by fibroblasts transformed to myofibroblasts is necessary during the initial phase of post-MI remodeling [31]. Therefore, our finding that HeC did not induce myofibroblast transformation suggest that the cardioprotective potential of HeC-treated fibroblasts may be limited. 

Several molecular pathways can induce the migration of CFs [33,34,35], some of which induce proliferation and transformation as well. However, as migration without accelerated proliferation was also described [23,36], the connection between these mechanisms is uncertain. miRNA-induced changes in gene expression have also been implicated in fibroblast migration [37,38]. Therefore, high-throughput transcriptomic investigations could uncover the molecular mechanisms of HeC-induced migration. 

As EVs play role in cardioprotective mechanisms [27], we expected that HeC modulate the release of mEVs by NRCFs. Here, we found that HeC tended to decrease the number of mEVs released by NRCFs, the cardioprotective role of which could be explained by a recent study which shown that EVs secreted by activated CFs impaired endothelial functions [39].

As not only the amount, but the composition of mEVs can be affected by HeC, we investigated whether the secretome of HeC-treated NRCF affects naïve fibroblasts or ECs. We found that HeC NRCF supernatant did not activate naïve NRCFs. This finding shows that HeC may have only a limited CF-derived effect on remote cardiac areas as compared to other conditioning interventions, such as nitrogen- and isoflurane-conditioning, which induced EV-mediated fibroblast migration [40]. Similarly, the supernatant of HeC NRCFs did not enhance the angiogenic potential of ECs. However, since leukocyte-derived inflammatory factors are needed for the proangiogenic effect of fibroblasts [41], which may manifest only days after MI [42], further investigations in complex systems with diverse cell types or in vivo studies are needed to assess the effect of the secretome or EVs obtained from HeC NRCFs.

In conclusion, we discovered increased fibroblast migration after HeC that may contribute to its beneficial role in cardiac repair. However, this effect was not transferable to remote cells. Although these findings demonstrate cellular mechanisms and potential benefit of HeC in post-MI repair, further studies are needed to evaluate its translational potential.

## 4. Materials and Methods

### 4.1. Isolation of Neonatal Rat Cardiac Fibroblasts (NRCFs)

NRCFs were isolated from hearts of 1–3 day-old male *Wistar* rats (Toxi-coop ltd., Budapest, Hungary). Briefly, pups were disinfected with 70% ethanol and sacrificed by cervical dislocation. Hearts were rapidly removed and were placed in ice-cold phosphate buffered saline (PBS) (Gibco, Paisley, UK). Ventricles were separated from atria and were minced and transferred into 0.25% trypsin (Gibco, Paisley, UK) solution. Cardiac tissue was digested at 37 °C for 25 min with agitation every 5 min. The resulting cell suspension was centrifuged for 15 min at 250 rcf at 4 °C and the supernatant was then removed. Cells in the pellet were resuspended in DMEM (Corning Inc., New York, NY, USA) supplemented with 10% fetal bovine serum (FBS) (Corning Inc., New York, NY, USA) and pre-plated for 90 min. Medium was collected to isolate NRCFs. NRCFs were passaged at 75% confluence using 0.5% trypsin/0.53 mM EDTA (Thermo Fisher Scientific, Waltham, MA, USA). The cell culture after the 3rd passage was discarded [43].

### 4.2. Handling of HUVEC-TERT2 Cell Line

HUVEC-TERT2 (Evercyte GmbH, Vienna, Austria, CHT-006-0008) were cultured on a 0.1% gelatin (Merck KGaA, Burlington, MA, USA)-coated surface using endothelial cell growth basal medium (Lonza Group Ltd., Basel, Switzerland) supplemented with human endothelial growth factor, hydrocortisone, bovine brain extract (BBE), ascorbic acid, FBS from Lonza SingleQuots Kit (Lonza Group Ltd., Basel, Switzerland), and 20 µg/mL G418 solution (Roche Diagnostics, Basel, Switzerland). Cells were kept at 37 °C in a 5% CO_2_/95% air environment and subcultivated according to the vendor’s protocol, when cells reached 80% of confluence.

### 4.3. Helium Conditioning of NRCFs 

NRCFs at 75–90% confluence were used for conditioning. Medium was removed and cells were washed with Hanks balanced salt solution (HBSS, Corning Inc., New York, NY, USA). Then, 200 µL/cm^2^ of glucose- and FBS-free growth medium (PAN-Biotech GmbH, Aidenbach, Germany) was added to the flasks, which were put into plastic bags with a loosened cap. The plastic bag was filled with 95% helium + 5% CO_2_ gas mixture five times to expel all the oxygen. Flasks were sealed in bag and put into the incubator at 37 °C for 1 h. After 1 h, flasks were withdrawn from the plastic bag and returned to normal growth conditions (5% CO_2_/95% air, 37 °C) for 1 h. The 1 h with helium/CO_2_ mixture and 1 h normal condition was repeated a total of 4 times. After the 4th cycle of conditioning, culturing medium was resupplied with 1 g/L glucose (Merck KGaA, Burlington, MA, USA) and left in normal growing conditions for 40 h. For control, the same culturing medium was used, but cells remained at normal growing conditions during the whole procedure. 

When NRCF supernatant was used for HUVEC-TERT2 angiogenesis or migration assay, FBS- and BBE-free HUVEC-TERT2 growth medium was used during the conditioning procedure instead of NRCF growth medium. 

### 4.4. Medium Extracellular Vesicles (mEVs) Isolation 

The supernatant of NRCFs was removed from the flasks and mEVs were isolated using differential centrifugation. Cell supernatants were centrifuged at 300 rcf for 10 min at 4 °C. Then supernatants were centrifuged at 2500 rcf for 5 min at 4 °C and then at 13,500 rcf for 40 min at 4 °C. Pellets were re-suspended in 200 µL PBS for Western-blot, 100 µL PBS for Flow cytometry, and DLS or in 200–1500 µL PBS for NTA. For flow cytometry and DLS, two additional centrifugations were performed at 13,500 rcf for 5 min at 4 °C followed by the exchange of supernatant with PBS.

### 4.5. Cell Migration Assay of NRCFs

NRCFs were seeded in 24-well plates pre-coated with 10 µg/mL fibronectin (Thermo Fisher Scientific, Waltham, MA, USA). For seeding of the cells, growth medium was removed from the flask and NRCFs were washed with HBSS (Corning Inc., New York, NY, USA). Cells were then mobilized with 0.5% trypsin/0.53 mM EDTA and incubated for 3–6 min at 37 °C. Trypsin solution was neutralized by DMEM supplemented with 20% FBS (Corning Inc., New York, NY, USA) and cells were collected to conical centrifuge tube. Cells were counted using a hemocytometer and a cell suspension equal to 100,000 cells was moved to each well. Growth medium with 20% FBS was added to each well. Cells were incubated on plate for 6–16 h at 37 °C in cell incubator, then the medium was removed from the wells and cells were washed with HBSS and growth medium was added into the wells. Plate was incubated at 37 °C in cell incubator until confluence. Sixteen hours before the assay, medium was removed, cells were washed and growth medium with 1% FBS was added to the wells. After incubation, the cell layer was scratched using a 200-µL pipette tip at a 30° angle. Cell debris was removed by washing the wells with HBSS. Finally, cells received growth medium with 1% FBS or supernatant medium of HeC or CTRL NRCFs supplemented with 1% FBS and incubated at 37 °C in cell incubator. Two pictures from each well were taken at 0 h, 8 h, and 24 h after the scratch using phase contrast microscope (Leica Microsystems, Wetzlar, Germany, UK) with 5× magnification and images were analyzed quantitatively using ImageJ (http://rsb.info.nih.gov/ij/ (accessed on 14 May 2018)). For each image, the area of the scratch was measured and the percentage of gap closure was calculated.

### 4.6. Myofibroblast Transformation Analysis by Quantitative Polymerase Chain Reaction (qPCR)

NRCFs were conditioned and lysed in RNAZol RT solution (Merck KGaA, Burlington, MA, USA). Total RNA isolation, cDNA (complementary deoxyribonucleic acid) synthesis, and qPCR measurement was performed as described earlier [44]. In brief, DNA and proteins were precipitated by nuclease-free water (Acros Organics, Geel, Belgium) and 4-bromoanisole (Sigma-Aldrich, St. Louis, MO, USA). Then, RNA was precipitated with isopropanol (Sigma-Aldrich, St. Louis, MO, USA) and pellets were washed with ethanol (VWR International, Fontenay-sous-Bois, France) before resuspension in nuclease-free water. cDNA synthesis was performed by Sensifast cDNA synthesis kit (Bioline; London, UK) according to the manufacturer’s protocol. Measurements were performed on a LightCycler^®^ 480 Real-Time PCR System (Roche Diagnostics, Basel, Switzerland) using LightCycler^®^ RNA Master SYBR Green I reagent (Roche Diagnostics, Basel, Switzerland) with primers presented in Supplementary Table S1. For control, Vimentin was used as a common fibroblast marker. Fold change was calculated using the ΔΔCt calculation method according to Schmittgen and Livak 2008 [45].

### 4.7. Myofibroblast Transformation Analysis by Western Blot

WB was performed as described earlier [46]. NRCFs were lysed in radio-immunoprecipitation assay buffer (RIPA; Cell Signaling Technology, Danvers, MA, USA) containing protease inhibitor (Amresco, Solon, OH, USA) and homogenized with sonication. Samples were centrifuged for 10 min at 12,000 rcf at 4 °C. Protein concentration in the samples was measured using a bicinchoninic acid (BCA) assay kit (Thermo Scientific, Waltham, MA, USA). For the analysis of isolated mEVs, isolates were mixed with 10× RIPA buffer in 1:10 dilution right before use. For the myofibroblast transformation assay, 6 µg of protein was used, and for mEV analysis, 30 µL of each sample was used. Antibodies are presented in Supplementary Table S2. For control, Vimentin was used as a common fibroblast marker. Signals were visualized using enhanced chemiluminescence kit (ECL, Bio-Rad, Hercules, CA, USA) or super ECL kit (Bio-Rad, Hercules, CA, USA) by Chemidoc XRS+ (Bio-Rad, Hercules, CA, USA). Band intensities were measured using Image Lab software (Bio-Rad, Hercules, CA, USA). α-SMA and Vimentin were normalized to DDR2, and TGF-β was normalized to GAPDH.

### 4.8. Cell Migration Assay of HUVEC-TERT2

HUVEC-TERT2 was seeded onto a 24-well plate and grown until confluence. Then 16 h before the assay, the medium was changed to growth medium supplemented with 1% FBS and 0.25% BBE. After incubation, the confluent cell monolayer was scratched from the surface using a 1000-µL pipette tip and growth medium from HeC NRCF or CTRL NRCF supplemented with 1% FBS and 0.25% BBE was added to the cells. Pictures from each well were taken in two defined positions at 0 h and 8 h using a phase contrast microscope (Leica Microsystems, Wetzlar, Germany). Images were analyzed using ImageJ software by a blinded analyzer. For each image, the area of the scratch was measured, and the percentage of gap closure was calculated. In each experiment, images from the same well were analyzed individually and the average of the gap closure values was calculated and treated as a single data point.

### 4.9. Tube Formation Assay of HUVEC-TERT2

NRCFs were conditioned with helium as described above. The supernatant was collected and supplemented with 5% FBS and 0.25% BBE. HUVEC-TERT2 culture was trypsinized, pelleted, and resuspended in supernatants from NRCFs. Ibidi angiogenesis µ-slides (Ibidi GmbH, Gräfelfing, Germany) were coated with Matrigel basement membrane (Corning Inc., New York, NY, USA) according to the producer’s protocol and 8000 cells/well were seeded. Images were taken 6 h after incubation using phase contrast microscope (Leica Microsystems, Wetzlar, Germany, UK) and analyzed using the automated ImageJ Angiogenesis Analyzer macro [47], without post-processing of the images. The number of master junctions was used for the quantification of the nodes, mean mesh size was used for the quantification of meshes, and Mesh index was used for the quantification of mean tube length.

### 4.10. Nanoparticle Tracking Analysis

mEV samples were isolated as described above and analyzed by Zeta View PMX 110 (Particle Metrix, Meerbusch, Germany) NTA machine. First, the machine was calibrated using 100-nm polystyrene beads according to the manufacturer’s protocol. Samples were diluted in PBS to have 50–300 particles in a view of sight. At least 1 mL of sample was injected into the machine and automated measurement was acquired with 11 positions throughout the measurement cell, with two cycles of readings at each position. Readings for which the software recommended exclusion were excluded from the final evaluation.

Instrument parameters were set as: temperature of 25 °C, sensitivity of 75, frame rate of 7.5 frames per second, shutter speed of 100. Post-acquisition parameters were set as: minimum brightness 20, minimum size 5 pixels, maximum size 1000 pixels. Results were multiplied by the dilution factor.

Particle concentration and modal particle size was used for the analysis of the samples. R programming language was used to visualize the data. The mean of all the measurements was calculated. Data were smoothed with Loess regression and visualized with +/− SEM.

### 4.11. Electron Microscopy of EV Samples

mEVs were isolated as described above. After 13,500 rcf centrifugation, the pellet was re-suspended in 1.5 mL 0.2 µm filtered PBS and centrifuged for 1 h at 100,000 rcf at 4 °C in a polypropylene ultracentrifuge tube. The sample was post-processed as described earlier [48]. In brief, the sample was fixed with 4% paraformaldehyde and postfixed in 1% osmium tetroxide (Taab, Aldermaston, UK). The pellet was dehydrated in graded ethanol, block stained with 1% uranyl acetate in 50% ethanol, and embedded in Taab 812 (Taab, Aldermaston, UK). After an overnight polymerization at 60 °C, the sample was sectioned to ultrathin sections using a Leica UCT6 ultramicrotome (Leica Microsystems, Wetzlar, Germany, UK) and examined with a Hitachi 7100 transmission electron microscope (Hitachi Ltd., Tokio, Japan). Images were taken by Veleta 2 k × 2 k MegaPixel side-mounted TEM CCD camera (Olympus, Tokio, Japan). Brightness and contrast were adjusted using Adobe Photoshop 7.0 (Adobe Systems Incorporated, San Jose, CA, USA).

## 5. Statistics

All results were statistically analyzed with GraphPad Prism 6 (GraphPad Software Inc., San Diego, CA, USA) using a Mann–Whitney *t*-test, except for the NTA analysis of mEVs concentration, where a paired Wilcoxon *t*-test was used. Differences were considered significant with *p* < 0.05.

## Data Availability

The data that support the findings of this study are available from the corresponding authors, M.B. (Monika Barteková) and Z.G., upon reasonable request.

## Supplementary Materials

The following are available online at https://www.mdpi.com/article/10.3390/ijms221910504/s1.

## Abbreviations

APC	Allophycocyanin
α-SMA	Alpha smooth muscle actin
BBE	Bovine brain extract
cDNA	Complementary deoxyribonucleic acid
CF	Cardiac fibroblast
CVD	Cardiovascular diseases
DLS	Dynamic light scattering
DMEM	Dulbecco’s modified eagle’s medium
EBM	Endothelial cell growth basal medium
EC	Endothelial cell
EDTA	Ethylenediaminetetraacetic acid
FBS	Fetal bovine serum
FC	Flow cytometry
HBSS	Hank’s balanced salt solution
HeC	Helium conditioning
HUVEC-TERT2	Immortalized human umbilical vein endothelial cells
IHD	Ischemic heart disease
mEVs	Medium extracellular vesicles
MI	Myocardial infarction
NRCF	Neonatal rat cardiac fibroblast
NTA	Nanoparticle tracking analysis
PBS	Phosphate buffered saline
PE	Phycoerythrin
RIPA	Radio-immunoprecipitation assay
RNA	Ribonucleic acid
qPCR	Quantitative polymerase chain reaction
TEM	Transmission electron microscopy
