# Supplementary material for: Helium Conditioning Increases Cardiac Fibroblast Migration Which Effect Is Not Propagated via Soluble Factors or Extracellular Vesicles
Source: Int J Mol Sci. 2021 Sep 29;22(19):10504. doi: 10.3390/ijms221910504 (PMC8508629; doi:10.3390/ijms221910504)
Supplement: Supplementary file 1 [file ijms-22-10504-s001.zip › ijms-1375983-supplementary.pdf]

Supplementary methods and Figures

Dynamic Light scattering

DLS was performed on Malvern Zetasizer Nano ZS 90 series device (Malvern Instruments, England) with a He–Ne laser (633 nm) and 90° collecting optics. DLS measures the hydrodynamic diameter of extracellular vesicles on basis of their Brownian motion while the DLS peak position and width provide the size and dispersion of the sample, respectively. Measurement took place in 12mm square disposable polystyrene cuvette at 25°C.

Flow cytometry measuring of viability of cells

Measurements were carried out on FACSCalibur flow cytometer (BD, San Jose, CA, USA) on the day of the staining. Forward (FSC) and side scatter (SSC) parameters were set in linear scale, and threshold was set on the FSC parameter. Cell gating was accomplished by propidium iodide (PI) viability staining (Figure S1 A,B). CellQuestPro software (BD, San Jose, CA, USA) was used for the acquisition and analysis. Data were also analyzed and presented using Flowing Software 2.5.1 (Turku Bioscience Centre, Turku, Finland).

Flow cytometry analysis of mEVs

Medium EVs were separated from the cell culture supernatant by differential centrifugation as described. Annexin V-APC (allophycocyanin), DDR2-Alexa488 (R&D Systems, FAB25381G) and Thy1-PE (Phycoerythrin) (SantaCruz Biotech, sc-19614) staining were used for the identification of mEVs. The „Direct Immunofluorescence Staining of Cells Using a Lyse/No-Wash Procedure" protocol of BD Biosciences was adapted for the staining of exofacial molecules of separated mEVs. Differential detergent lysis was used for the validation of mEV measurements. Those events that did not disappear in the presence of 0.1% Triton-X 100 were rejected from analysis as described by György et al. [37].

Measurements were carried out on FACSCalibur flow cytometer (BD, San Jose, CA, USA) on the day of the staining. Forward (FSC) and side scatter (SSC) parameters were set in logarithmic scale, and threshold was set on the SSC parameter. mEV gating was accomplished by preliminary standardization experiments using Megamix-Plus SSC beads (Biocytex, France) (Figure S3 A,B). CellQuestPro software (BD, San Jose, CA, USA) was used for the acquisition and analysis. Data were also analyzed and represented by using of Flowing Software 2.5.1 (Turku Bioscience Centre, Turku, Finland).

Filtration isolation of EVs

H9C2 cells were grown as described earlier [38]. Cells were kept in FBS-free medium for 24 hours, then supernatant was centrifuged for 10 minutes at 300 rcf at 4°C, and then for additional 5 minutes at 2,500 rcf at 4°C. Then supernatant was filtered through mixed cellulose esters membrane (Merck KGgA, Burlington, USA, 0.8µm) or polyethersulfone (Pall, New York, USA, 0.8µm) membrane or remained unfiltered. Then samples were centrifuged for 6 hours at 100,000 rcf at 4°C and re-suspended in RIPA buffer. Samples were used for WB as described.

Supplementary figures

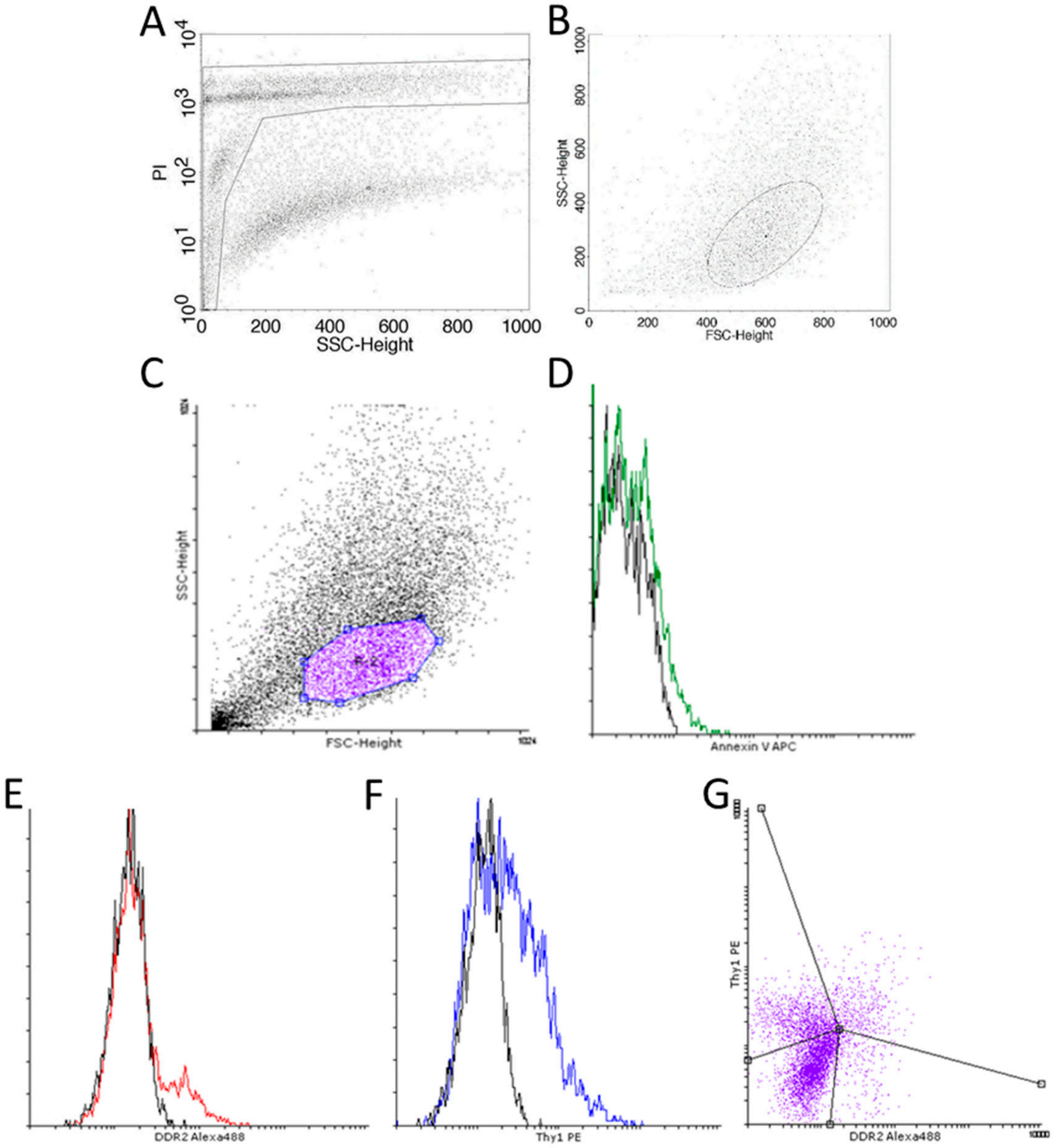

Figure S1: Analysis of NRCF cells with FC for typical NRCF markers (A). Propidium iodide (PI) is a membrane impermeant dye that is excluded from viable cells but easily penetrates the damaged, permeable membranes of non-viable cells. PI emission was detected on FL2 channel. The SSC vs FL2 dot plot was used for the definition of dead cells (R1 gate). PI positive dead cells were removed from analysis, and PI negative (viable) cells were displayed on the FSC-SSC dot plot (B) and were gated around for the definition of living cells (R2). Viability was also determined by Annexin V staining. The exofacial appearance of phosphatidylserine (PS) residues on the cell membrane is an early event in apoptosis. Annexin V has a strong, Ca^{2+}-dependent affinity for PS and therefore it can be used as a probe for detecting apoptosis. Annexin V staining was performed to validate the living cell gate. (C) The representative FSC vs SSC dot plot shows the "living cell" gate which was defined by PI staining. (D) The representative overlay histogram shows the Annexin V binding of cells inside the "living cell" gate. (E) The representative overlay histogram shows the DDR2 binding of cells inside the "living cell" gate. (F) The representative overlay histogram shows the Thy1 binding of cells inside the "living cell" gate. (G) Co-expression of Thy1 and DDR2 was also determined. The representative dot plot illustrates the immunophenotype of cells.

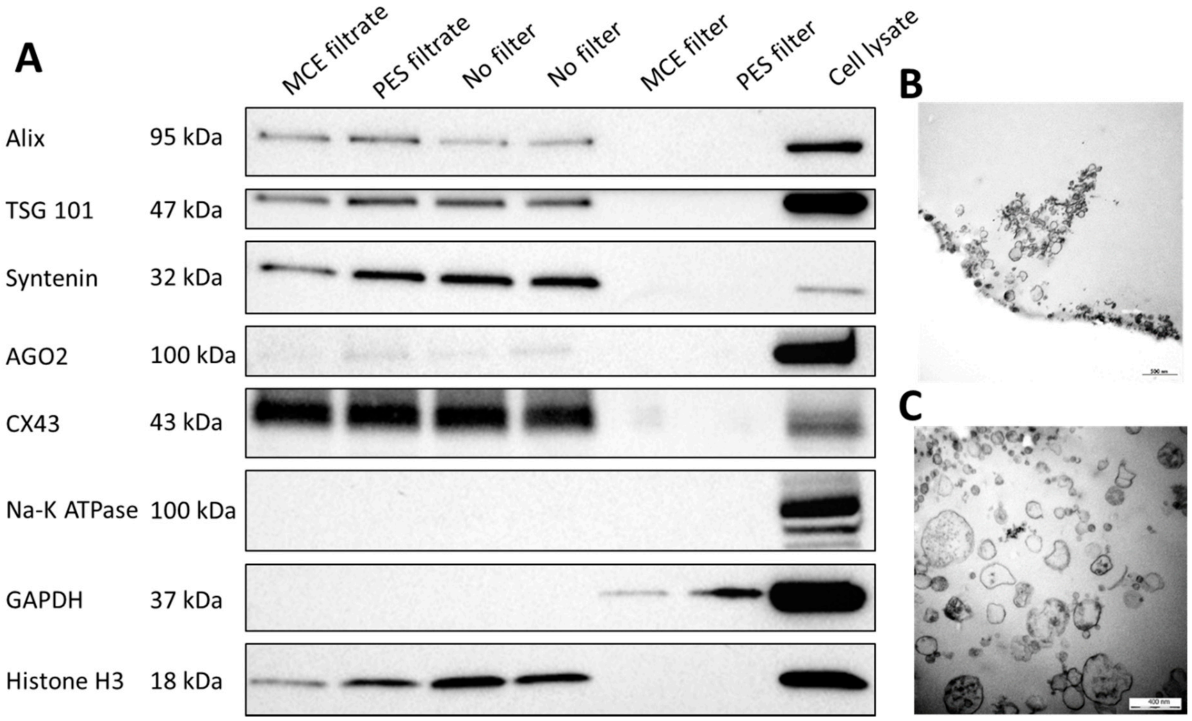

Figure S2: Filtration does not increase EV purity but may reduce yield (A) Western blot results of EV isolated by differential centrifugation with mixed cellulose esters (MCE) filtration, polyethersulfone (PES) filtration, or without filtration (No filter), and filters after used for EV isolation (MCE filter and PES filter) (B) Electron microscopic image of MCE filtered EVs (C) Electron microscopic image of non-filtered EVs.

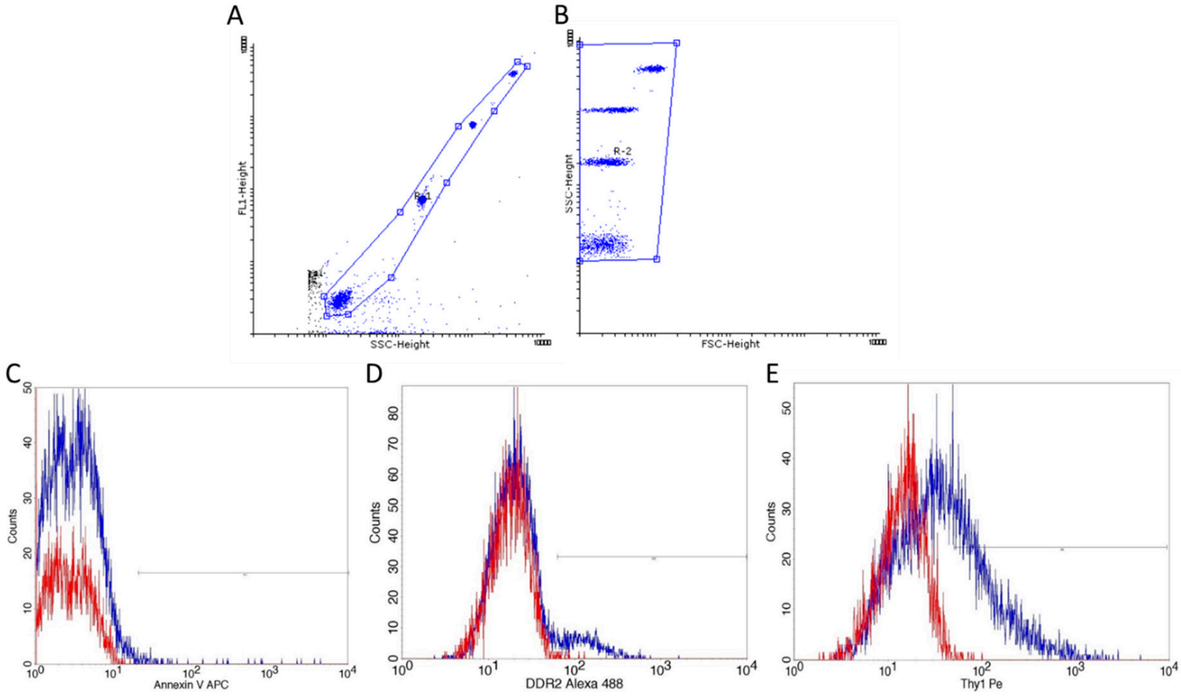

Figure S3: SSC beads (Biocytex, France). The fluorescence of the beads (FL1 signal) was displayed against the side scatter (SSC) because the SSC parameter is proportional to the size of the detected particles (A). The SSC vs FL1 dot plot was used for the definition of beads within the 0.2 µm – 2 µm size range (R1 gate). Bead populations within the R1 gate were displayed on the FSC-SSC dot plot (B) and were gated around for the definition of mEV

gate (R2). Representative overlay histograms show the exofacial phosphatidylserine, DDR2 and Thy1 expression of mEVs (C, D, E). Blue curves represent the fluorescence intensity of stained mEVs inside the mEV gate. Red curves show the detected fluorescence after TritonX 100 detergent lysis. Separated mEVs showed low phosphatidylserine expression mEVel, as was performed by AnnexinV staining (C). Only a little part of the mEVs expressed DDR2 (D), while the presence of Thy1 could be detected on mEVs, but the expression mEVel of this antigen was moderate (E).

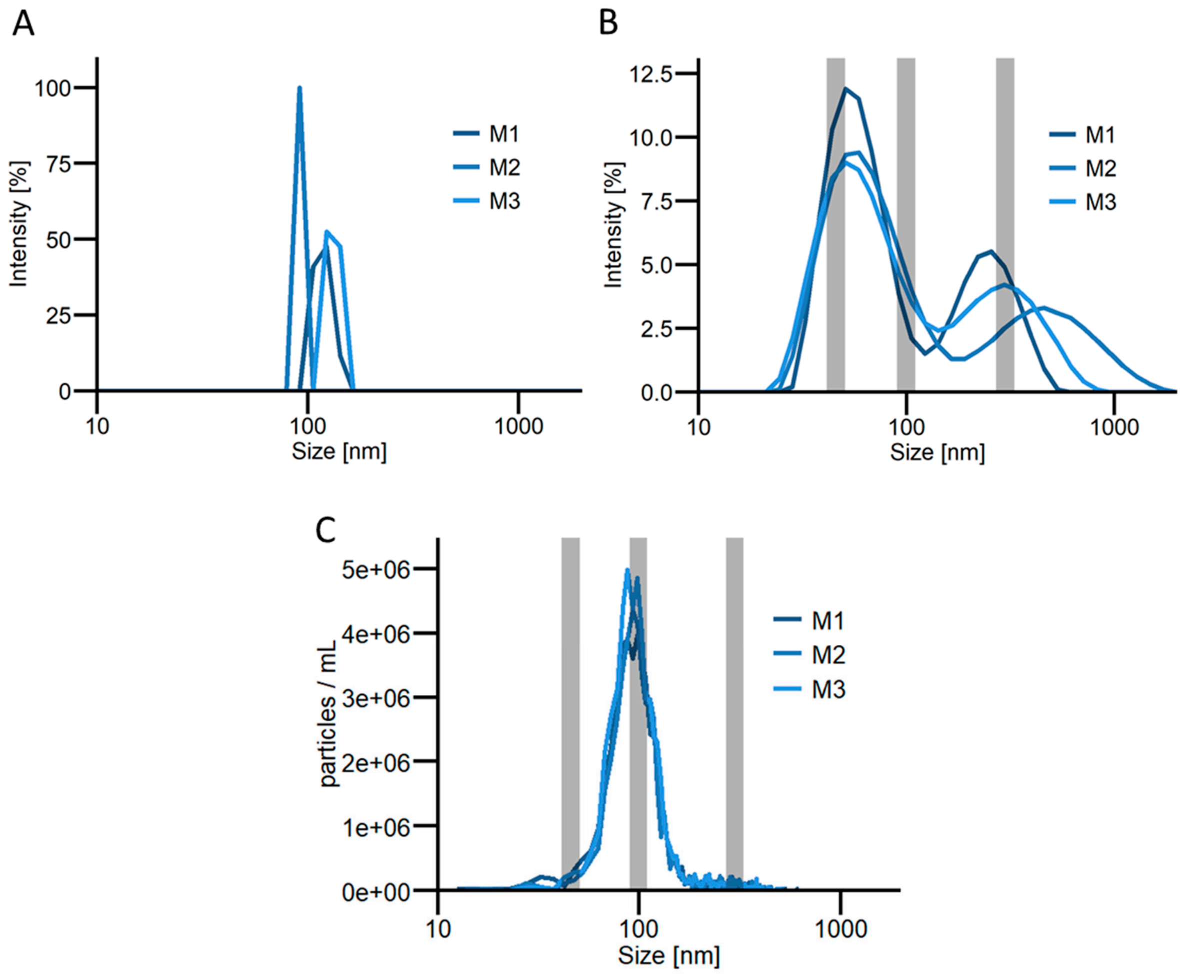

Figure S4: Characterization of DLS and NTA with calibration nanobeads; (A) DLS analysis of isolated mEVs returns only a single peak for every 3 repeats. (B) DLS resulted with two peaks for the mixture of nanobeads (46 nm, 100 nm, 300 nm) with variable modal particle size corresponding to the 300 nm beads (C) NTA resulted a single peak at 100 nm with high reproducibility for mixture of nanobeads (46 nm, 100 nm, 300 nm)

Supplementary Tables

Table S1: List of the antibodies used for the western blot analysis of fibroblast to myofibroblast transformation or the qualitative analysis of NRCF mEVs.

Gene	Gene symbol	Vendor	Ref No.
Alpha-smooth muscle actin	α-SMA	Abcam	ab7817
Annexin A1	ANXA1	Abcam	ab214486
Discoidin domain-containing receptor 2	DDR2	Cell Signaling Technology	12133
Glyceraldehyde 3-phosphate dehydrogenase	GAPDH	Cell Signaling Technology	D16H11
Organelle detection western blot cocktail		Abcam	ab133989
Transforming growth factor beta	TGF-β	Abcam	ab92486
Vimentin	Vimentin	Cell Signaling Technology	D21H3

Table S2: List of the primers used for the qPCR analysis of fibroblast to myofibroblast transformation

Gene	Gene symbol	Primer sequences	Annealing temperature
alpha-smooth muscle actin	α-SMA	CTATTCCTTCGTGACTACT<br>ATGCTGTTATAGGTGGTT	60
collagen type 1 alpha 2	Col1α2	CCTCTGGTGATCCTGGCAAAC<br>TCACCGGGAAGACCCCTTTC	62
fibroblast activation protein	FAP	TTGAAGGTTACCCTGGAAGAA<br>TGGCTTTGTAGCTGAAACTTG	60
Glyceraldehyde 3-phosphate dehydrogenase	GAPDH	TACCAGGGCTGCCTTCTCTTG<br>GGATCTCGCTCCTGGAAGATG	60
Interleukin 1β	IL1β	TCAGGAAGGCAGTGTCACTCATTG<br>ACACACTAGCAGGTCGTCATCATC	60
Interleukin 6	IL6	TCTCTCCGCAAGAGACTTCCA<br>ATACTGGTCTGTTGTGGGTGG	60
Transforming growth factor beta	TGF-β	ACAGGGCTTTCGCTTCAGTG<br>GTCCAGGCTCCAAATGTAGGG	62
Vimentin	Vimentin	ACCAGAGACGGACAGGTGA<br>CTTGCGCTCCTGAAAACTGC	60
